# The suitability assessment for land territorial spatial planning based on ANN-CA model and the Internet of Things

**DOI:** 10.1016/j.heliyon.2024.e31237

**Published:** 2024-05-14

**Authors:** Zhaoliang Nie

**Affiliations:** a School of Resources, Environment and Architectural Engineering, Chifeng University, Chifeng, 024000, China; b Key Laboratory of Land Space Planning and Disaster Risk Prevention and Control in Chifeng City, Chifeng 024000, China

**Keywords:** Land spatial planning, Internet of Things (IoT), Artificial neural network, Cellular automaton model, Urban suitability assessment, Cellular automaton

## Abstract

This work aims to utilize Internet of Things (IoT) technology and the Artificial Neural Network - Cellular Automaton (ANN-CA) model to analyze the construction of indicators for territorial spatial planning and urban development suitability assessment. Firstly, the IoT technology is introduced, and its application potential in land planning is explored. Using the IoT technology, various data related to land use are collected, and these data are transmitted and summarized through IoT equipment to form a data base. Based on the collected data, the ANN-CA model and the “dual assessment” concept are employed to establish an indicator system for urban development suitability assessment, encompassing permanent basic farmland, ecological redlines, and current built-up areas. Through the combination of these two models, the future land use situation can be predicted more accurately. The trained model is evaluated, including simulation accuracy, error analysis, Kappa coefficient and other indicators. Compared with the actual data, the accuracy and credibility of the model are verified. Finally, according to the prediction results of the model, the land use situation is analyzed and interpreted to provide decision support for urban planning and development. The research results show that the combination of IoT technology and ANN-CA model can effectively analyze the evolution law of land use and the suitability of urban development. Through the reasonable setting and processing of model parameters and data, people can get high accuracy land use prediction results, which provides important reference and support for urban planning and sustainable development. The suitability for urban development within County M exhibits noticeable spatial disparities, with the central region being more suitable for development while the peripheral regions are relatively less favorable. This work provides valuable guidance for decision-makers and researchers in the field of territorial planning, and promotes orderly urban development and sustainable prosperity.

## 1 Introduction

### 1.1 Research background and motivations

In the current rapid global urbanization context, the importance of land territorial spatial planning has become increasingly prominent. Urbanization brings about various challenges, including rational land resource utilization, environmental protection, and infrastructure development [[1], [2], [3], [4]]. However, traditional planning methods face issues such as insufficient data acquisition, model complexity, and prediction accuracy [[5], [6], [7]]. Exploring an innovative strategy to assisting land territorial spatial planning becomes critical to effectively solve these difficulties.

The rapid emergence of the Internet of Things (IoT) presents new opportunities for addressing these issues. IoT facilitates richer and real-time data acquisition and enhances the accuracy of monitoring and analyzing spatial systems [8,9]. However, applying IoT data to the field of land territorial spatial planning requires the establishment of effective models and analytical methods. In this context, researchers have explored models that combine the Artificial Neural Network (ANN) with Cellular Automata (CA) to simulate and evaluate urbanization processes with greater granularity [[10], [11], [12]]. It is now feasible to better comprehend the dynamics of urban evolution, resource distribution, environmental changes, and more by exploiting real-time data gathered from IoT and combining models such as ANN and CA.

The research motivation of this work can be summarized as follows. Firstly, the challenges brought by urbanization. With the rapid development of global urbanization, the process of urbanization has brought a series of challenges, including the rational use of land resources, environmental protection, infrastructure construction and so on. These challenges need to be solved by effective regional spatial planning methods [13,14]. Secondly, the problem of traditional planning methods. There are some problems in the traditional land regional spatial planning method, including insufficient data acquisition, model complexity and prediction accuracy. These problems limit the effectiveness of planning [15,16]. Thirdly, the opportunity of the IoT. The rise of IoT technology provides new opportunities. IoT technology not only enriches the sources of data acquisition, but also improves the accuracy of monitoring and analysis of space systems [17]. Fourthly, combining artificial intelligence (AI) and CA. In order to better understand the trends of urban evolution, resource distribution and environmental changes, researchers began to explore a model combining ANN with CA [18]. This combination is expected to improve the complexity and accuracy of the model. Finally, a new intelligent method. The ultimate goal of the work is to explore and construct an innovative method to deal with the complexity and challenges of urbanization in an intelligent and scientific way [19]. By integrating technologies such as IoT, ANN and CA, the research aims to provide scientific basis and strategic guidance for future sustainable urban development.

Therefore, the research motivation of this work is to meet the challenges brought by rapid urbanization, and to provide more intelligent and comprehensive methods for regional spatial planning and urban development by using emerging technologies and models.

The goal of this work is to investigate a method for land territorial spatial planning and suitability assessment based on the ANN-CA model. This work is closely related to the United Nations Sustainable Development Goals (SDGs), especially in supporting sustainable cities and communities (SDG 11). By optimizing land use and promoting the intellectualization of urban planning, this work helps to achieve the balance among efficient use of resources, environmental protection and economic development, which are the core contents of SDGs. This work also suggests an urban development suitability evaluation method that takes into account more aspects, with the goal of providing planners with an innovative and intelligent strategy to dealing with the complexities and issues that come with urbanization. A deeper understanding of the emerging patterns inside urban systems can be accomplished by integrating technologies such as IoT, ANN, and CA, providing scientific underpinnings and strategic direction for future sustainable urban development.

The importance of the work lies in its various contributions to the realization of the United Nations SDGs. Firstly, by accurately evaluating the suitability of land use, urban planners can be guided to formulate more scientific and reasonable land use strategies, thus improving land use efficiency and reducing the damage to the natural environment. Secondly, this work helps to protect basic farmland and ecological red line areas by predicting the trend of urban expansion, which is very important for maintaining ecological balance and biodiversity. In addition, the work also emphasizes the role of transportation network and infrastructure construction in promoting sustainable development, which is in line with the goal of SDG 9 (industry, innovation and infrastructure). Through these measures, the work not only supports sustainable urban development, but also provides practical ways to realize economic growth, social inclusion and environmental sustainability. To sum up, this work provides a powerful decision support tool for urban planning and development by comprehensively applying advanced technologies and models. This is not only helpful to realize the rational utilization of land resources and the efficient management of urban space, but also of great significance to promote the realization of the United Nations SDGs. The practical application of the research results will promote the orderly development of the city, improve the quality of life of residents, and protect and improve the ecological environment at the same time, which reflects the firm commitment and practical actions to the goal of sustainable development.

### 1.2 Research objectives

The work aims to explore and establish an innovative approach for land territorial spatial planning and adaptive assessment by leveraging the combination of IoT, AI models, and CA. The goal is to truly grasp the subtle links between urban growth and land utilization by leveraging the benefits of IoT in data gathering and real-time monitoring, along with ANN training. CA aids in simulating the dynamic evolution of urban systems while taking into account numerous aspects that influence spatial patterns and development trends. Furthermore, the research goal includes the creation of a complete assessment system to analyze the viability of urban growth within land territorial spatial planning. The novelty of the research lies in using the advantages of data acquisition and real-time monitoring of IoT, combining with ANN and CA, deeply understanding the complex relationship between urban development and land use, and considering the influence of various factors on spatial pattern and development trend. It is helpful to simulate the dynamic evolution of urbanization process and resource distribution more accurately, and improve the complexity and accuracy of the model. The research aims to provide scientific basis and intelligent planning methods for sustainable urban development in the future. By integrating technologies such as IoT, ANN and CA, people can better understand the evolution law of urban system, provide strategic guidance for urban decision makers, and promote the orderly and sustainable development of cities.

## 2 Literature review

The application of IoT in land territorial analysis has become one of the current research hotspots. Mugiyo et al. (2021) focused on land suitability analysis in the agricultural domain. By reviewing various land suitability analysis methods, they found that using big data and IoT could enhance the accuracy and reliability of land suitability analysis [20]. Cheng and Wang (2021) employed techniques like texture mapping and panoramic imagery for image modeling. They utilized IoT and wavelet analysis to design an IoT architecture for urban land use efficiency management, enabling monitoring and management of urban land use [21]. Zhao et al. (2022) emphasized challenges and opportunities in urban development and introduced an innovative densely populated IoT approach to address urban planning and resource management issues [22]. IoT can provide real-time data and in-depth insights into various aspects of land use and spatial dynamics within a region. Hence, it held a transformative impact on understanding and managing land resources, environmental factors, and urban development trends [[23], [24], [25]] However, the research in the above literature also has limitations. For example, the research results in Ref. [26] are limited by the unavailability of classified datasets, which is a huge challenge. The research results in Ref. [27] are limited to the application of IoT in big cities, and it remains to be considered whether the research structure will be affected under the harsh environment in remote areas.

With the rapid advancement of neural networks, their advantages in land spatial analysis are gradually evident. Saputra and Lee (2019) employed the ANN-CA model to simulate and predict land use and land cover changes in North Sumatra Province, Indonesia. The anticipated outcomes were more accurate than the actual maps, demonstrating that the ANN-CA model may give credible future land cover projections [28]. e Silva et al. (2020) utilized a multi-layer perceptron neural network for dynamic land cover modeling. They used ANN to study land cover changes in the Taperoá River Basin in northeastern Brazil, achieving favorable accuracy and assessment metrics [29]. Zhou et al. (2022) aimed to improve urban land subsidence prediction and proposed a multi-factor neural network algorithm-based land subsidence prediction method. The results demonstrated high accuracy and reliability in predicting urban land subsidence [30]. Neural networks have shown their effectiveness in capturing complex spatial relationships and patterns [31,32].

Kumari et al. (2019) paid attention to the processing of secure streaming data generated by different devices, especially the processing of big data in the IoT environment, and discussed the architecture and security methods required for each stage of secure streaming processing. They emphasized the main threats and risks in big data analysis, such as denial of service, malware and phishing attacks [33]. Their research provided a useful background and idea for the data processing and security of the IoT. Kumari et al. (2018) paid attention to multimedia big data, studied the uniqueness and complexity of multimedia big data in the application of the IoT, and developed a comprehensive multimedia big data classification, abstracting it into a new process model reflected in the IoT [34]. Their study provided inspiration for the methods and models of multimedia data processing in the IoT. Kumari and Tanwar (2021) put forward a security demand response management scheme in smart grid system named Q-SDRM, which was used for home energy management. The scheme adopted reinforcement learning and Ethereum blockchain to reduce energy consumption and energy cost [35]. This research provided an interesting reference for the work in smart grid and energy management. Patel et al. (2022) introduced the relationship between smart grid and renewable energy collection, emphasized the key role of renewable energy collection in managing the gap of electricity demand, and proposed a recommendation system for residential solar photovoltaic system based on artificial intelligence. The main goal of this method was to accurately predict the energy generation based on solar photovoltaic [36]. They provided a useful reference for the work in the field of solar energy. These studies provided useful background information on safe streaming data processing, multimedia data processing, household energy management and renewable energy. The research in these fields provided valuable inspiration and ideas for discussing the regional spatial planning and adaptability evaluation method based on ANN-CA model. Similarly, the research in the above-mentioned literature also has limitations. In the research of reference [37], CNN was introduced. However, whether the training performance of CNN was affected by the system environment or not needs to be solved to truly realize the high-precision analysis of big data. The research in Ref. [38] was to introduce networking technology into enterprise intelligent management, but whether different environments or different memories had an impact on hardware facilities in the process of hardware use needs further analysis. Reference [39] aimed to develop a structural framework for the IoT to adopt obstacles, but the source of obstacles was very single, which cannot prove the universality of the results. Reference [40] discussed the potential problems of medical care based on the IoT, the market obstacles for medical professionals and patients to adopt the IoT, confidence and acceptability, privacy and security, interoperability, standardization and reward, data storage, and control and ownership. However, whether these problems can be effectively solved in actual scenes remains to be verified.

With the continuous development of information technology, all kinds of sensors play a vital role in land planning, which can sense the situation of land use in real time and provide accurate data support for planning. Remote sensing sensors can obtain a wide range of land use information through satellite remote sensing, aerial remote sensing and other means, and can provide high-resolution image data for monitoring land use changes and identifying different types of land cover. Geographic information system sensors are used to collect geographic information data, including land boundaries, topography, land ownership and other information, and provide spatial data support for land planning. Meteorological sensors measure meteorological factors such as temperature, humidity and wind speed, which helps to evaluate the climate suitability of land and provide reference for agricultural and ecological protection planning. Soil sensors are used to monitor soil moisture, nutrient content, PH value and other indicators, and provide data support for agricultural use of land and vegetation restoration. In order to effectively use the IoT technology, it is very important to process, store and manage the data collected by sensors. The sensor transmits the collected data to a data acquisition node, such as a base station or a data collector, through wireless communication technology. Traditional communication methods include Wi-Fi, Bluetooth, Zigbee and so on, which are used to transmit data to the local area network. The collected data can be stored in a local server or a cloud server. Cloud storage has high scalability and flexibility, and can realize real-time backup and remote access of data. Commonly used cloud service providers include Amazon Web Services (AWS), Microsoft Azure, Google Cloud, etc. The collected raw data may need to be cleaned, filtered and processed to extract effective information. Data processing algorithms can be run on local servers or cloud servers, and data analysis and pattern recognition can be realized by using machine learning, deep learning and other technologies. Through the API interface or data transmission protocol provided by the cloud platform, the processed data is exported to the cloud database. These data can be used by planners, policy makers and researchers for further analysis and utilization.

Ramson et al. (2021) proposed a land use monitoring scheme based on LoRaWAN technology [41]. They used LoRaWAN wireless sensor network to monitor land use types and changes in real time, and transmitted the data to the cloud server for storage and analysis. Through this scheme, the accurate management and visual monitoring of land resources in the process of urbanization were realized. Chen et al. (2022) designed a land quality monitoring system based on NB-IoT technology. They used NB-IoT sensors to monitor soil moisture, temperature, nutrient content and other indicators in real time, and uploaded the data to the cloud database to provide data support for agricultural production and land planning [42]. The research results showed that the system had good real-time performance and stability. Xing et al. (2023) proposed a dynamic monitoring method of urban land use based on satellite remote sensing and IoT technology [43]. They used satellite remote sensing images and urban sensor networks to monitor the changes of urban land use in real time, and used AI algorithms to analyze and predict the data. This method provided new ideas and technical means for urban land planning and management. Li et al. (2023) developed a land ecological environment monitoring system based on wireless sensor network [44]. They used wireless sensor networks to monitor the ecological environment quality of land in real time, including air quality and water quality. Through this system, the land ecological environment can be monitored and managed in time, which provided a scientific basis for ecological protection and land planning. Pamula et al. (2022) proposed a dynamic monitoring and evaluation method of urban land use based on IoT technology [45]. They used the IoT sensor network to monitor the urban land use status in real time, and combined the geographic information system and AI technology to dynamically evaluate and predict the land use. The results showed that this method can effectively monitor urban land use change and provide scientific basis for urban planning and management. The application of IoT technology in the field of land planning had broad development prospects. Through the collection, storage and analysis of sensor data, it can provide important support for the rational utilization of land resources and the sustainability of urban development.

The existing research has made important contributions in exploring land planning suitability assessment based on IoT technology and ANN-CA model, but there are also some gaps worthy of attention. Firstly, although the potential application of IoT technology in land planning is mentioned in the existing literature, there is a lack of in-depth discussion on the specific data collection, processing and management processes. For example, the discussion on security and privacy protection in data processing and storage is limited, and these issues are very important in practical applications. In addition, the existing research has failed to fully consider the applicability of IoT technology under different environmental conditions, especially the reliability and stability in remote areas or harsh environments. In addition, the specific application cases and parameter setting of ANN-CA model in land planning are also lacking, and there is a lack of in-depth evaluation of its practical application effect. In view of the shortcomings of the above existing research, this work puts forward a land planning suitability evaluation method based on IoT technology and ANN-CA model, and fills the gaps in the existing research. Firstly, this work discusses the data collection, processing and management process of IoT technology in land planning in detail, especially the issues of data security and privacy protection, thus providing a feasible solution for practical application. Secondly, the application scenarios and parameter settings of ANN-CA model are fully discussed, and the reliability and effectiveness of ANN-CA model in land planning are verified through a comprehensive evaluation of its application in practical cases. In addition, this work fully considers the applicability of IoT technology under different environmental conditions, and puts forward corresponding countermeasures, thus ensuring the stability and operability of the method. Generally speaking, this work fills some important gaps in the existing research and provides a comprehensive and feasible solution for land planning suitability evaluation based on IoT technology and ANN-CA model. Through the in-depth research and practical application of IoT technology and ANN-CA model, this work provides important theoretical support and technical guidance for the further development and practice of land planning.

## 3 Research methodology

### 3.1 The application of IOT and the ANN-CA model in land territorial spatial planning

IoT can provide abundant data and intelligent support for land territorial spatial planning, thereby optimizing decisions related to resource utilization, environmental management, and urban development [[46], [47], [48]]. Data gathering and monitoring, geographical analysis and decision support, and smart city planning are all examples of how IoT is being used in land territorial spatial planning. IoT provides data, analytical capabilities, and intelligent support to land territorial spatial planning, assisting in the optimization of urban development, resource usage, and environmental management. As a result, cities are being pushed in a more intelligent and sustainable path [[49], [50], [51], [52]].

**Fig. 1:**
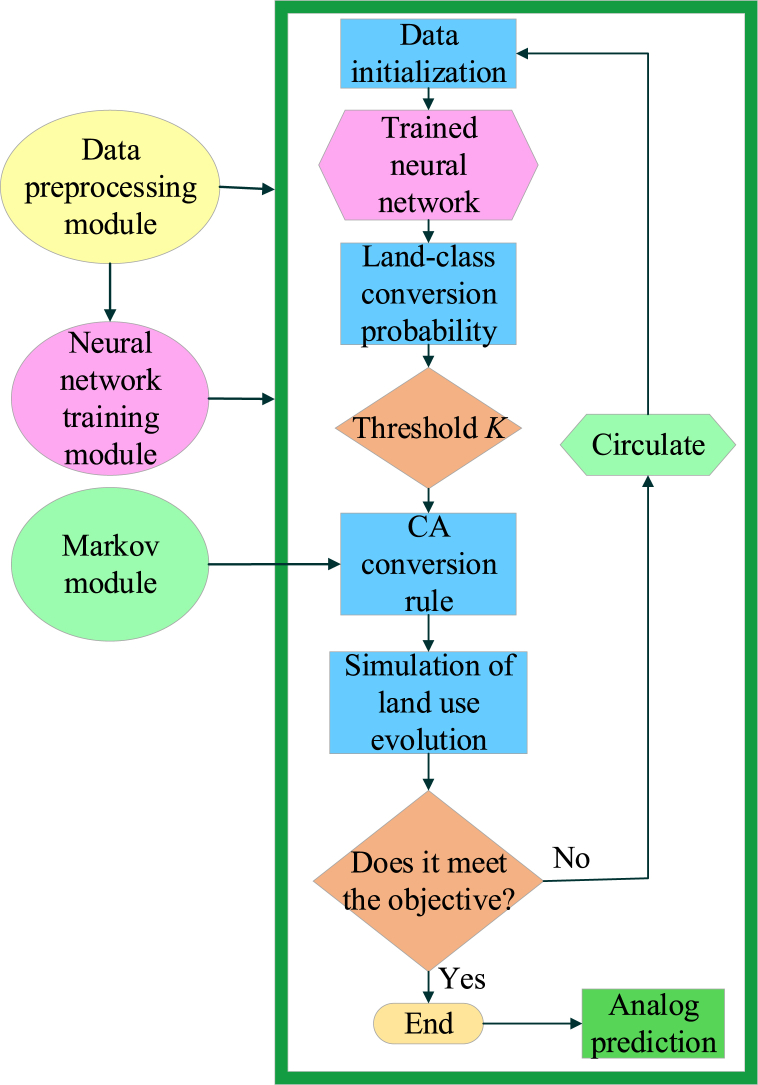
The framework of the ANN-CA Model.

The ANN-CA model, a combination of ANN and CA, is a computational model used for simulating and analyzing complex spatial systems. It aims to achieve more accurate and comprehensive spatial system simulations and predictions [53,54]. Typically, the ANN is responsible for feature extraction and prediction of input data, while the CA simulates the dynamic evolution process of the spatial system [55,56]. The structural framework of the ANN-CA model mainly consists of five modules: data preprocessing, Markov prediction, ANN training, model calibration, and prediction, as illustrated in Fig. 1 [[57], [58], [59]].

Fig. 1 shows the structural framework of ANN-CA model, which consists of five main modules: data preprocessing, Markov prediction, ANN training, model correction, and prediction. The data preprocessing module is mainly responsible for obtaining the original data and standardizing it. Markov prediction module analyzes historical land use changes through Markov chain and predicts the total amount of future land use units. ANN training module includes neural network design, random sampling of training data, network training, and result testing. The model correction module corrects the period of ANN-CA model by using the data of land use status in real years, the total land use predicted by Markov and the trained neural network, and simulates the land use status in real years. The prediction module infers the future urban land use change trend and simulates the future land use situation according to the land use data of real years, the total land use predicted by Markov and the trained neural network combined with the periodic operation model with clear correction process [[60], [61], [62]]. Firstly, the ANN training module is introduced in detail. The data supplied into the ANN describe the limits and driving forces in the evolution of urban land use. During ANN training, all factors input to the input layer are stored in the characteristics of each simulated unit. The chance of a simulation unit changing land use type at time t is the consequence of the interplay of that unit's consistent properties. In other words:

(1) $$X\left(k,t\right)={\left[x_{1}\left(k,t\right),x_{2}\left(k,t\right),\cdots ,x_{n}\left(k,t\right)\right]}^{T}$$

$x_{i}\left(k,t\right)$ represents the *i*-th variable of unit *k* at simulation time *t*, which also signifies the influence factor of the *i*-th input neuron. *T* stands for transpose, and *n* represents the number of variables. After preprocessing, these variables are passed to the input layer of ANN. This is to measure the conversion probability of land use types. After preprocessing the data, the input layer receives standardized data, transmitting it to the hidden layer. The signal received by the *j*-th neuron in the hidden layer is:

(2) $${net}_{j}\left(k,t\right)=\sum \omega_{i,j}x_{i}\left(k,t\right)+c_{j}\left(k,t\right)$$

${net}_{j}\left(k,t\right)$ and $c_{j}\left(k,t\right)$ represents the signals the *j*-th neuron receives in the hidden layer from pixel *k* at training time *t*. $\omega_{i,j}$ is the weight value between the input layer and the hidden layer. This process is used to extract features and calculate signals. The input layer transfers data to the hidden layer, and after receiving the input, the hidden layer generates a corresponding response value. The response function of the hidden layer is:

(3) $$f\left(x\right)=\tan\;sig\left(x\right)=\frac{e^{x}-e^{-x}}{e^{x}+e^{-x}}$$

Equation (3) represents the hidden layer response function in ANN, and the hyperbolic tangent sigmoid function is used to generate the response value. The hidden layer outputs the response values to the output layer, and the response value of the output is:

(4) $$f\left({net}_{j}\right)=f\left[\sum \omega_{i,j}x_{i}\left(k,t\right)+c_{j}\left(k,t\right)\right]$$

Equation (4) describes the signal transmission from the hidden layer to the output layer and the calculation of the response value. The response values are transmitted to the output layer, and the data received by the output layer is:

(5) $${net}_{u}=\sum \omega_{i,j}f\left({net}_{j}\right)+c_{u}\left(k,t\right)$$

Equation (5) describes the calculation process of the output layer in ANN, which is similar to the calculation of the hidden layer, but this is the calculation of the output layer. ${net}_{u}$ and $c_{u}\left(k,t\right)$ represent the signals the u-th neuron receives in the output layer from pixel *k* at training time *t*. After the output layer receives the response value data, the excitation function is:

(6) $$g\left(x\right)=\log\;sig\left(x\right)=\frac{1}{1-e^{-x}}$$

Equation (6) represents the excitation function processing of the output values of ANN, which maps the output values to the range of [0,1], and these output values represent the conversion probability of land use types. The output values processed by the excitation function are between [0,1], and these output values are the transformation probabilities of land use types trained by neural networks, and their mathematical expressions are as follows:

(7) $$p\left(k,t,u\right)=\frac{1}{1+e^{-{net}_{u}\left(k,t\right)}}$$

(8) $${net}_{u}\left(k,t\right)=\sum \omega_{i,j}f\left({net}_{j}\right)+c_{u}\left(k,t\right)=\sum \omega_{i,j}f\left[\sum \omega_{i,j}x\left(k,t\right)+c_{j}\left(k,t\right)\right]$$

The above equation ${net}_{u}\left(k,t\right)$ includes the following sets of independent variables: land use type, economic development level, population density, traffic convenience and topography. These factors are standardized by data preprocessing module and then input into ANN model. Considering the randomness of real land use change and the weak influence of neural network training output value on land use change, a random disturbance variable and a threshold value *O* are added to the model to control the output of model conversion probability and iteration time. The mathematical expression of random disturbance variable b is:

(9) $$b=1+{\left[-\ln\;\left(rand\right)\right]}^{r}$$

rand is a random number of [0,1]. r is a parameter that controls the value range of b. Equation (9) introduces how to control the output of model transition probability by introducing random disturbance variable *b* and threshold value *O*. This mechanism introduces some randomness to better simulate the uncertainty in the real land use change.

The basic elements of CA module include cell, state, neighborhood and land use evolution rules. The cells represent grid data in a cellular space with a grid size of 30 m*30 m. This size is selected based on the satellite image resolution (such as Landsat 8 data) used. Each cell has a unique identification code and a corresponding set of attributes. States consider transitions from other land uses to urban land use types and transitions among various urban land use types within the city. Neighborhood density influences the probability of land use change transitions. The used cellular neighborhood radius is 3, and the extended Moore's neighborhood is 7 * 7. Land use evolution rules input the driving factors of land use evolution into neural network for training, and obtain the rules parameters of CA model to simulate land use change. The expression of the land use evolution rules reads:

(10) $$f:R_{i}^{t+1}=f\left(R_{i}^{t},R_{N}^{t},V_{i}^{t},V_{N}^{t},b_{i}^{t},O,W\right)$$

$R_{i}^{t+1}$ and $R_{i}^{t}$ represent the states of cell *i* at time *t*+1 and time *t* respectively. $R_{N}^{t}$ and $V_{N}^{t}$ are the combinations of the neighborhood states of cell *i* at time *t* and its attribute state acquired from the neural network. $V_{i}^{t}$ represents the comprehensive attribute state of cell *i* obtained from the neural network at time *t*. $b_{i}^{t}$ is the random perturbation to the land use transition probability of cell *i* at time *t*. O stands for the threshold of the land use transition probability set in the model. W is a control variable artificially introduced into the CA model [[63], [64], [65]].

The model correction mainly adopts Lee-Sallee shape index to compare the spatial distribution similarity between the simulated land use data and the present situation, and mainly calculates the ratio of the spatial intersection and union area between the simulation results and the actual data units used to test the model, which is expressed as:

(11) $$L=\frac{A_{0}\cap A_{1}}{A_{0}\cup A_{1}}$$

$A_{0}$ and $A_{1}$ represent the simulated land use data and the real land use status data used to test the simulation results respectively. The prediction module assumes that the land use change in the study area is in a stable development state during the study period. After the correction module determines the threshold and random parameter r with the highest simulation accuracy, it executes the workflow of the correction module to predict the future land use change [66,67]. The integrated ANN-CA model is useful in areas such as land territorial spatial planning and suitability assessment since it can take into account all facets of the spatial system.

In addition, the communication cost and calculation cost of ANN-CA model are evaluated. The communication cost usually depends on the data transmission mode, data size and communication protocol, while the calculation cost usually depends on the complexity of the model, the use of computing resources and running time. The communication cost is estimated by evaluating the data transmission volume, transmission frequency and communication protocol in the model, and then the communication cost is estimated, usually in the form of data transmission cost and bandwidth usage. The calculation cost is estimated by evaluating the calculation resource requirements of the model, including 10.13039/501100002857CPU, memory, storage, etc., and then estimating the calculation cost of the model, usually in the form of hardware/cloud service rental fees, power costs, etc. When building the model, each module is independent, so that people can understand the framework of the model more intuitively, but it also leads to complicated model operation and increased cost.

### 3.2 The construction of a suitability assessment index system

In the process of selecting driving factors and suitability evaluation indicators, the research results of many scholars are referenced. For example, Wang et al. (2022) emphasized the importance of land resources utilization, environmental protection and infrastructure construction in the process of urbanization. These studies provide a basic understanding of urbanization challenges and planning needs [68]. Meanwhile, Tobias et al. (2020) discussed the importance of spatial planning in the protection of cultivated land, and pointed out that with the continuous growth of cities and infrastructure, its effectiveness is often questioned [69]. In addition, they also pointed out that there was a lack of methods to evaluate the effectiveness of spatial planning. In Switzerland, the revision of the national spatial planning law in 2014 provided a new starting point for stricter regulations on urban development. The study evaluated whether the new regulations can better protect special high-quality cultivated land from the impact of transforming into urban areas through land use suitability model and land use scenario simulation. It was found that according to the revised planning method, the potential consumption of high-quality cultivated land for new urban areas is 6 times smaller than that predicted by the urban development trend in the past 25 years. However, the scenario simulation showed that there will still be more high-quality cultivated land transformed into urban areas, and it may be difficult to protect cultivated land according to the degree required by Swiss high-quality cultivated land protection policy. They put forward a method to evaluate spatial planning measures. However, in order to keep the amount of cultivated land up to the level required for agricultural self-sufficiency and food safety, these planning measures need to be strictly implemented. Based on these studies, it is recognized that under the background of rapid urbanization, land spatial planning should not only consider the efficiency of land use, but also give consideration to ecological protection and sustainable development. Therefore, a set of indicators that can comprehensively reflect these needs is selected. Specifically, the following indicators are considered.

1. Land resources: It includes altitude, slope, etc. These indicators directly affect the cost and difficulty of land development.
2. Water resources: The area close to water resources is very important for the life and industrial activities of urban residents.
3. Geographical advantages: It includes the accessibility of traffic arteries and the proximity of traffic centers, these factors affect the connectivity and attractiveness of the region.
4. Traffic network density: A high-density traffic network helps to support urban expansion and economic growth.
5. Basic farmland and ecological red line: These are important resources to protect the regional agricultural foundation and ecological balance.
6. Existing built-up area: It is very important to consider the existing infrastructure and urban structure for urban renewal and expansion.

The specific reason for choosing indicators in this work is based on the comprehensive impact of these factors on urban development. By giving different indexes different weights, people can evaluate the suitability of urban development in different regions, thus providing decision support for urban planners. In addition, the concept of “double evaluation”, that is, evaluating the suitability of urban development by establishing an index system, has not been fully discussed in the existing literature, which provides a new perspective for our research. In terms of comprehensive and applicability analysis, the selected indicators are not only scientific and reasonable, but also have practical application value. Through consultation with urban planning and land management experts, the effectiveness of these indicators in actual planning decision-making is verified. The comprehensiveness of these indicators makes the evaluation system adapt to the needs of different regions and different stages of development, and their applicability ensures that the evaluation results can provide practical guidance for decision makers.

The framework known as “dual assessment” in land territorial spatial planning serves as the main foundation for research on the appropriateness assessment of urban development. It adds assessments of permanent basic agriculture, ecological redlines, and the existing built-up regions to the initial single-factor evaluations of land resources, water resources, and locational benefits. Each evaluation factor is assigned values for different levels and categorized into five grades. Ultimately, the suitability of urban construction is classified into five levels. Table 1 presents the indicators for suitability assessment in urban spatial planning [[70], [71], [72]]. The suitability evaluation index system proposed in Table 1 is to evaluate the suitability of urban development. This system considers several key factors to ensure that land use planning can meet the various needs of urban development. Areas at lower altitudes are usually more suitable for construction because they are easier to develop and visit. Slower slopes are conducive to building construction and infrastructure construction, so areas with smaller slopes are considered more suitable for development. Areas close to water resources are usually more suitable for development, because water resources are vital to the life and industrial activities of urban residents. Areas close to major traffic arteries are more suitable for development, because convenient transportation can promote economic activities and the flow of people. Being close to transportation hubs, such as railway stations and airports, can improve regional connectivity and attractiveness. Areas located near the city center are usually more suitable for development, because these areas usually have higher economic activities and population density. Areas with high traffic network density are usually more suitable for development, because a good traffic network can support urban expansion and economic growth. Basic farmland is an important resource to ensure food security, and its protection is very important to maintain the agricultural base and ecological balance of the region. Ecological red line areas refer to those areas that have important ecological functions and need strict protection. In these areas, urban development is restricted to protect the ecological environment and biodiversity. The existing built-up areas are the land that has been developed and utilized, and the expansion and redevelopment of these areas need to consider the existing infrastructure and urban structure. These indicators are divided into different grades, and each grade corresponds to a different suitability score. By evaluating and weighting these indicators, a comprehensive suitability score can be obtained, thus providing decision support for urban planning and development. The purpose of this evaluation system is to ensure that land use planning can balance economic development, environmental protection and social needs and promote the sustainable development of cities.

**Table 1:** Indicators and levels of suitability assessment for urban spatial planning.

Primary indicators	Secondary indicators	Third level indicators	Levels	Level classification
Land resources	Elevation	/	5	≤300 m
			4	300–350 m
			3	350–400 m
			2	400–450 m
			1	>500 m
	Slope	/	5	≤3°
			4	3–8°
			3	8–15°
			2	15–25°
			1	>25°
Water resource	/	/	5	≤100 m
			4	100–200 m
			3	200–500 m
			2	500–1000 m
			1	>1000 m
Location advantage	Geographic conditions	Accessibility of traffic arteries	5	≤20min
			4	20–40min
			3	40–60min
			2	60–90min
			1	>90min
		Accessibility of transportation hubs	5	≤20min
			4	20–40min
			3	40–60min
			2	60–90min
			1	>90min
		Accessibility in the central urban area	5	≤20min
			4	20–40min
			3	40–60min
			2	60–90min
			1	>90min
	Traffic network density	/	5	≤0.2 km/km^2^
			4	0.2–0.4 km/km^2^
			3	0.4–0.7 km/km^2^
			2	0.7–1.5 km/km^2^
			1	>1.5 km/km^2^
Basic farmland	/	/	5	≤300 m
			4	300–500 m
			3	500–800 m
			2	800–1000 m
			1	>1000 m
Ecological redlines	/	/	5	≤300 m
			4	300–500 m
			3	500–800 m
			2	800–1000 m
			1	>1000 m
Current built-up area	/	/	5	≤50 m
			4	50–100 m
			3	100–200 m
			2	200–500 m
			1	>500 m

## 4 Experimental design and performance evaluation

### 4.1 Experimental materials

This work focuses on County M. Situated to the south of the Tropic of Cancer in the northern hemisphere, County M experiences a subtropical monsoon humid climate characterized by hot and rainy summers, as well as cool and dry winters. It is surrounded by mountains on all sides, possessing high-quality mountain and water resources and displaying distinct characteristics of a mountainous urban area. By the end of 2022, the county had a resident population of 112,000 and a regional gross domestic product of 12.3 billion yuan.

The data includes county-level image maps of M County, Digital Elevation Model (DEM) data, national land survey data, vector polygon data for permanent basic farmland, ecological redlines, and county administrative boundaries, road vector line data, and vector point data of big data Point of Interest (POI), like highway entrances and exits, government centers, and transportation passenger terminals. These data sources are obtained from geographic spatial data cloud websites, the natural resources department of M County, the Open Street Map (OSM) website, and the Baidu Maps website. In order to transform the data into a format recognizable by the ANN-CA model, normalization procedures are required for each involved dataset. The methods for normalization are as follows:

(12) $$x_{i}^{\prime }=\frac{x_{i}-x_{\min}}{x_{\max}-x_{\min}}$$

$x_{i}^{\prime }$ is the normalized data, $x_{i}$ represents the data for the specific factor, $x_{\min}$ is the minimum value of that data, and $x_{\max}$ is the maximum value of that data. Ultimately, the normalization process yields results typically ranging between 0 and 1. The model's simulation accuracy is assessed using the method of quantitative precision error testing:

(13) $$s=\frac{Q_{ij}-Q_{il}}{Q_{ij}}*100\%$$

s represents the accuracy error for the *i*-th land use type. $Q_{ij}$ and $Q_{il}$ respectively denote the actual total number of units and simulated total number of units for the i-th land use type. A smaller absolute value of s indicates a higher accuracy of the model's simulation. The Kappa coefficient is employed to assess spatial patterns, measuring the consistency between two images.

The Analytic Hierarchy Process (AHP) is employed to assign weights to each evaluation factor in urban spatial planning suitability assessment. Individual evaluation results are then averaged with their appropriate weights to produce suitability assessment results for urban spatial planning. After calculation, the weights of each index are as follows: permanent basic farmland: 0.25, ecological red line: 0.35, and existing construction land: 0.4. In this work, the consistency ratio (CR) is 0.04, which is lower than the conventional acceptance standard of 0.1, indicating that the weight distribution has good consistency. The above results show that the ecological red line has the most important influence on the suitability of urban development, followed by the existing construction land and finally the permanent basic farmland.

### 4.2 Experimental environment and parameters setting

**Fig. 2:**
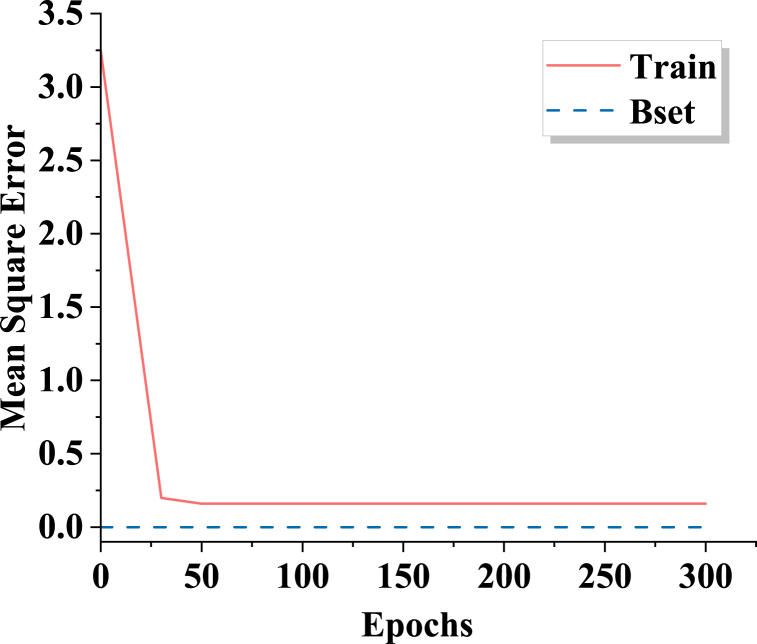
Results of ANN training.

The ANN-CA employs a 3-layer backpropagation neural network. The ANN architecture consists of an input layer with 13 neurons, a hidden layer with 10 neurons, and an output layer with 4 neurons. Overall, 10,000 randomly selected grid points are extracted from the 2010 land use status in County M to serve as training sample data. The model randomly chooses sample data for different land categories, such as agricultural land, forested land, water bodies, and urban areas. The neural network is trained for 300 iterations. Fig. 2 depicts the training results.

Fig. 2 illustrates that the ANN begins to converge after 30 iterations, with the training error curve flattening out. When it reaches 300 iterations, the error is 0.1597, meeting the requirement for basic accuracy. This neural network training outcome is stored and utilized for simulating the transformation rules of land use evolution in County M in 2020.

### 4.3 Performance evaluation

#### 4.3.1 The role of IoT technology in the simulation results

In this work, the IoT technology has played a vital role, especially in collecting real-time and more accurate land use data. By deploying IoT equipment, land use can be monitored in real time, and these data can be used for training and verification of simulation models. The following is a comparison table between the simulation results and the actual land use data monitored by IoT technology.

In Table 2, the difference between the simulation results and the IoT monitoring data is very small, which indicates that the simulation model has high accuracy. Especially for farmland and urban areas, the simulation results are very close to the actual monitoring data, and the conversion probability is close to 1, which shows that the model can predict the use changes of these land types well. Although the simulation model shows high accuracy, there are still some differences. For example, the IoT monitoring data in forest areas show more growth than the simulation results, which may be due to the failure of the model to fully consider some natural growth factors or small-scale illegal logging activities. In addition, the monitoring data of water area showed a slight decrease, which may be related to seasonal climate change or temporary human activities. By comparing with the actual land use data, it is proved that IoT technology has made remarkable contributions in improving the accuracy of simulation results. The real-time data provided by IoT technology enables the model to capture the actual changes of land use in time, thus improving the prediction ability of the model. In addition, IoT technology can also monitor small-scale or short-term changes that may be ignored by the model, providing valuable calibration data for the model. By comparing with the actual land use data, it is proved that IoT technology has made remarkable contributions in improving the accuracy of simulation results. The real-time data provided by IoT technology enables the model to capture the actual changes of land use in time, thus improving the prediction ability of the model. In addition, IoT technology can also monitor small-scale or short-term changes that may be ignored by the model, providing valuable calibration data for the model.

**Table 2:** Comparison between simulation results and IOT monitoring data.

Land use type	Simulation results (hectare)	IoT monitoring data (hectare)	Difference (hectare)	Conversion probability	Variation tendency
Farmland	5000	4980	−20	0.996	Decrease
Forest	1000	1020	+20	1.020	Increase
Water	1500	1490	−10	0.993	Stable
City	3000	3020	+20	1.007	Increase

#### 4.3.2 Accuracy assessment of the ANN-CA model

**Fig. 3:**
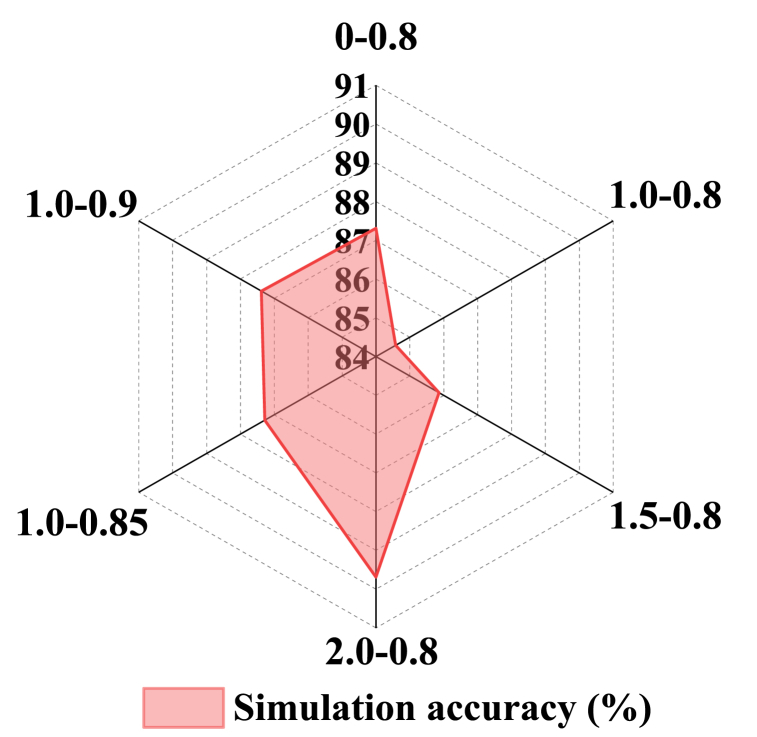
Accuracy of the ANN-CA model under different parameter combinations.

Different conversion probability threshold *O* values and random perturbation parameter *b* may influence the model's accuracy in simulating current land use. In order to determine the most suitable parameter combination for the ANN-CA model in the study area, six parameter combinations are established to simulate the land use situation in County M for the year 2020. The simulated land use map is then compared with the remote sensing interpreted land use data from 2020. Fig. 3 shows the influence of different parameter combinations on the accuracy of ANN-CA model through bar graphs. The horizontal axis represents different parameter combinations, and the vertical axis represents the simulation accuracy of the model. From the figure, with the change of transition probability threshold *O* and random disturbance parameter b, the accuracy of the model presents different results. Among them, the combination of parameters with *O* value of 0.8 and b value of 2 makes the simulation accuracy of the model the highest, reaching 89.69 %, which shows that this parameter combination is the best in this work.

#### 4.3.3 Analysis of predicted land use evolution

**Fig. 4:**
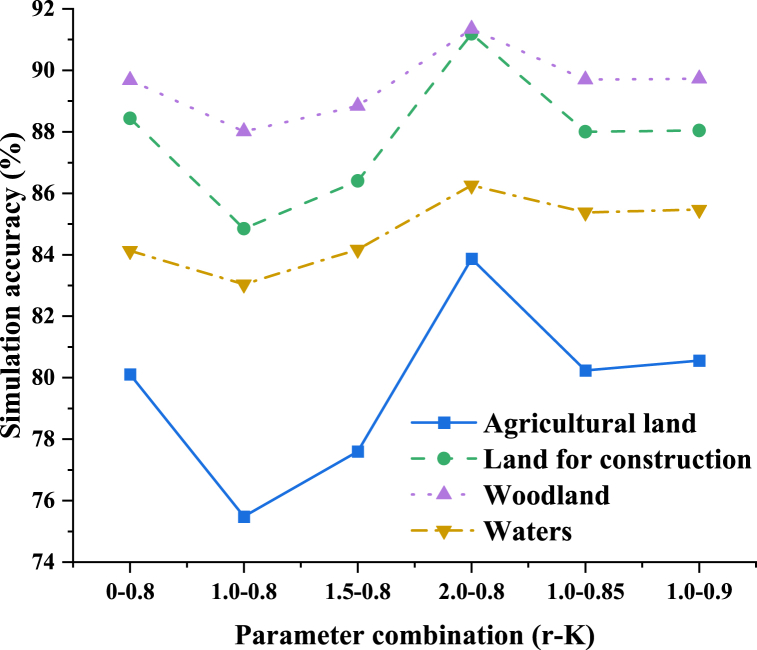
Simulation accuracy of different land categories under various parameter combinations.

Fig. 4 further refines the simulation accuracy of the model on different land types. The line chart in the figure shows the simulation accuracy of all kinds of land (such as agricultural land, forest land, water body, etc.), with the horizontal axis representing different parameter combinations and the vertical axis representing the simulation accuracy. It shows that under the combination of parameters with *O* value of 0.8 and b value of 2, the simulation accuracy of all kinds of land is high, which shows that the model can effectively predict the land use changes of different types.Therefore, the parameter combination with *O* at 0.8 and b at 2 is chosen to construct the model for predicting land use conditions in County M for the year 2030.

**Fig. 5:**
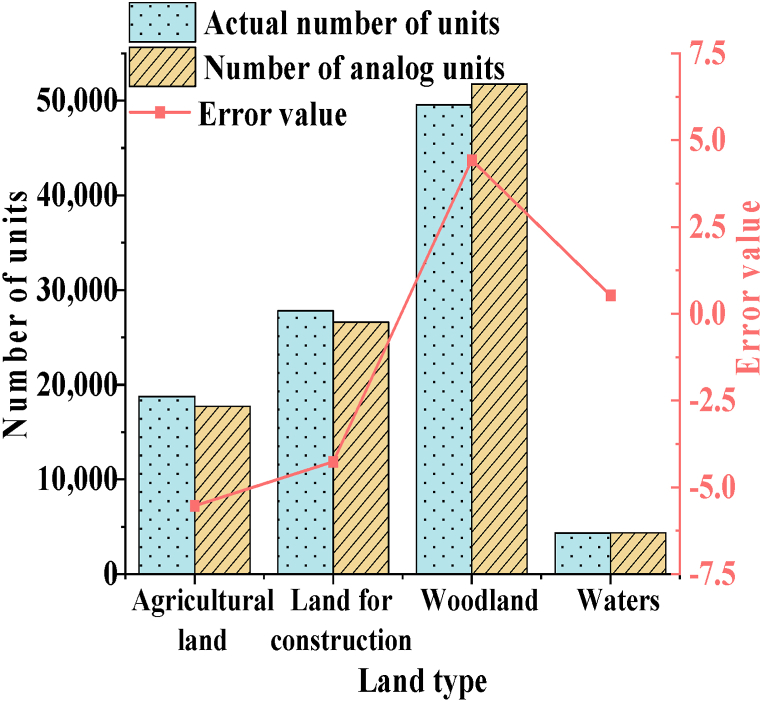
Simulation results of different land categories in County M for the year 2020.

Model accuracy has been verified. Quantity accuracy is examined using the unit counts of different land categories in County M for the year 2020. Fig. 5 illustrates the results.

**Fig. 6:**
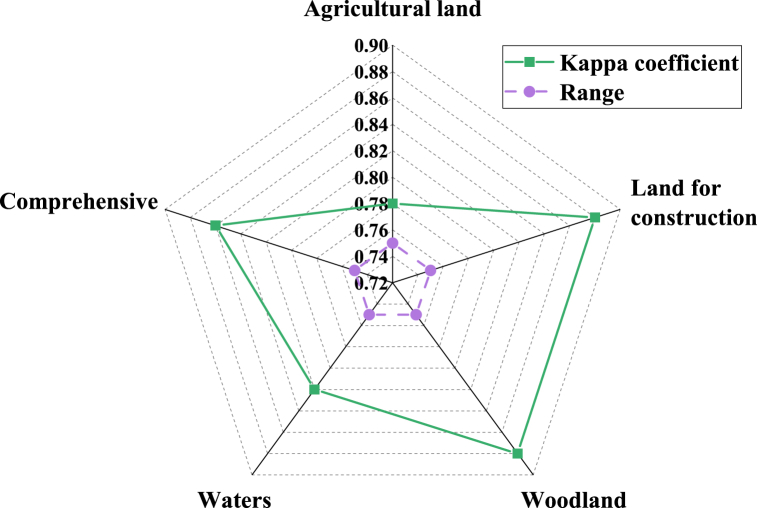
The Kappa coefficients for different types of land in County M in the year 2020.

Fig. 5 displays simulation error values for various types of land, all of which are smaller than 6 grid units. Among them, the smallest error value is 0.53 for water bodies, indicating that the model's prediction of land use in County M is reliable. Fig. 6 represents the Kappa coefficients for different types of land.

Fig. 6 suggests that the smallest Kappa coefficient among various types of land is 0.78 for agricultural land, and the comprehensive Kappa coefficient is 0.86. All types of land and the comprehensive Kappa coefficient are greater than 0.75. It indicates that the model has high accuracy and good effectiveness in simulating the land use change in M County in 2020 and has high credibility.

**Fig. 7:**
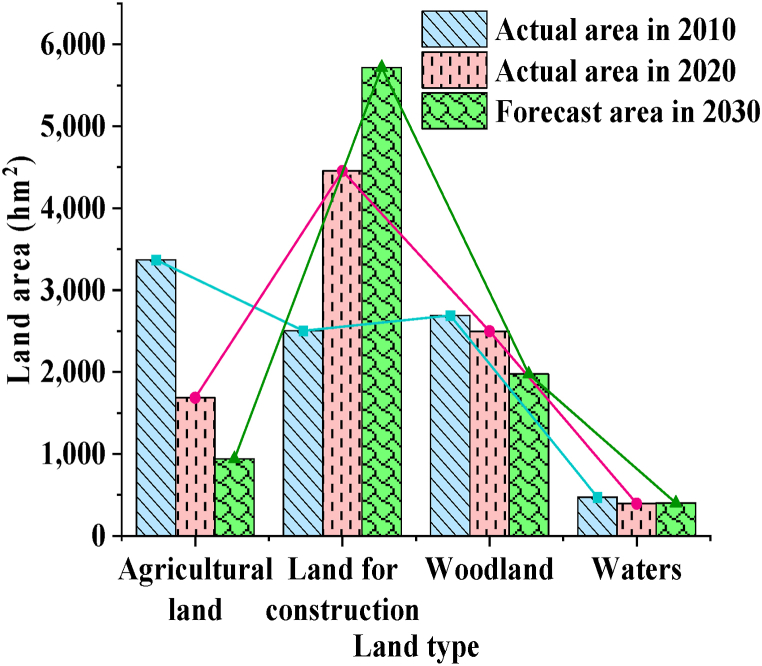
The land use changes in County M.

Then, according to the land use change from 2010 to 2020, using the determined parameter combination and neural network operation model, the land use prediction result of M County in 2030 is shown in Fig. 7.

Fig. 7 shows that the extent of each land type in County M has changed for the year 2030. Notably, development land continues to expand, while agricultural land and forested areas continue to shrink. The bodies of water remain pretty stable. The loss in agricultural land and rise in construction land between 2020 and 2030 are smaller than those between 2010 and 2020. The rate of land expansion for construction has also slowed marginally. These expected effects are consistent with the County M government's desire to improve the efficient and conservative use of construction land in accordance with urban development goals.

#### 4.3.4 The suitability assessment of land territorial spatial planning

**Fig. 8:**
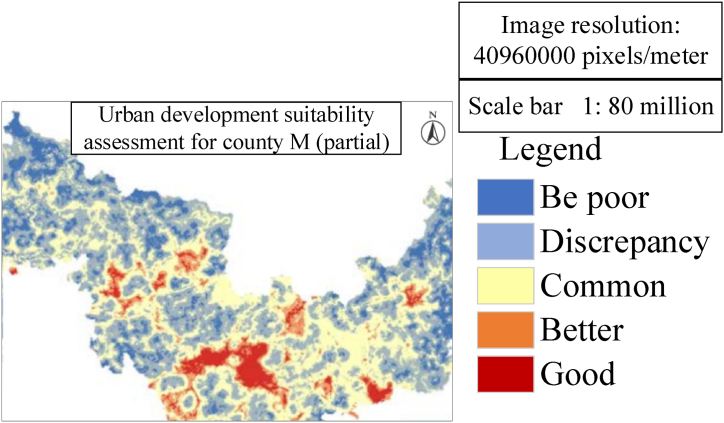
Urban development suitability assessment for County M (Partial).

**Fig. 9:**
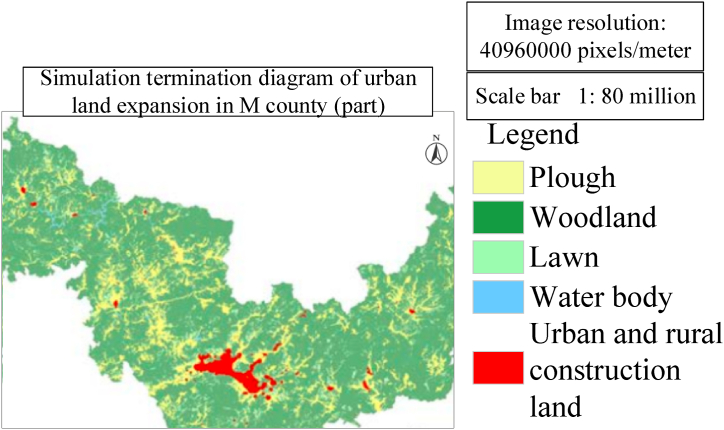
Simulation termination diagram of urban land expansion in M county (Part).

Fig. 8, Fig. 9 respectively show the simulation results of urban development suitability assessment and urban land expansion in the document. The connection between these two maps is that they jointly reflect the spatial distribution characteristics of urban development and the trend of land use change. Fig. 8 shows the results of the suitability evaluation of the urban development of County M, revealing that the central area of the city is more suitable for development, while the peripheral areas of the city are relatively unsuitable. This assessment is based on many factors, such as land resources, water resources, geographical advantages, traffic network density, basic farmland, ecological red line and existing built-up areas. These factors jointly determine the construction suitability grade of different regions and provide decision support for urban planning and development. Finally, the suitability for urban development in County M is evaluated based on the constructed urban suitability assessment index system. Fig. 8 presents a partial excerpt of the assessment results.

Fig. 8 reveals that the suitability for urban development exhibits significant regional variations within County M. The areas with favorable construction conditions are primarily concentrated in the central zone of the county town, while areas with less favorable conditions are concentrated at the outskirts of the county town, aligning with the developmental pattern of County M. The evaluation results are helpful for urban planners to identify and give priority to those areas that are more suitable for development, thus promoting the orderly and sustainable development of cities.

#### 4.3.5 Analysis of simulation results of urban expansion

The land use data of the simulated initial year is the land use data of M county in 2010, and the main comparison data is the land use data of M county in 2020. For the CA simulation of future towns, many iterations are needed to determine whether the unit is converted to ensure the accuracy of the results. Based on this, the number of iterations of this research simulation has been tested many times, including 300 times, 400 times and 500 times, and finally the end of urban and rural land growth for 500 iterations is determined, so the research results of 500 times are selected as the final urban expansion results. Setting conversion rules for each land, this model simulation study is mainly aimed at urban and rural construction land. Based on the principle of ecological protection and utilization, construction land cannot be converted into grassland and water land. Cultivated land cannot be converted into water and forest land. Grassland cannot be transformed into cultivated land, water body and forest land. Thus, the order and rules of transformation are set, and finally more reasonable simulation results can be obtained. The result is shown in Fig. 9. Fig. 9 shows the simulation results of urban land expansion based on ANN-CA model, which are consistent with the evaluation results of urban development suitability in Fig. 8. Fig. 9 shows that the expansion of urban and rural construction land is mainly concentrated in the urban center area, which is consistent with the core area assessed as suitable for development in Fig. 8. Meanwhile, Fig. 9 also shows that the speed of urban expansion has slowed down, which may be due to the low land suitability outside the central area of the city and the possible ecological protection and land use restrictions.

In Fig. 9, the total output area of urban and rural construction land is 5717.29 hm^2^, which is 1263.52 hm^2^ more than that of urban and rural construction land in 2020. The analysis trend of the simulation results is consistent with the prediction results, which further confirms the effectiveness of the ANN-CA model. Generally speaking, the connection between Fig. 8, Fig. 9 is that they both reveal the centralized trend of urban development and the rationality of land use change. The suitability assessment of urban development provides a scientific basis for land use change, and the simulation results of land expansion verify the accuracy and practicability of these assessments. The consistency of the two shows that the evaluation of urban development suitability by comprehensively considering various factors can effectively guide the actual land use planning and urban expansion, and promote the orderly and sustainable development of the city.

### 4.4 Discussion

In recent years, the integration of IoT into the field of neural networks has become increasingly common. Ali et al. (2022) examined the use of IoT in urban planning and healthcare. To address security and privacy concerns in IoT networks, they developed a unique technique based on group theory and deep neural networks for detecting intrusions. The effectiveness of the proposed strategy was evaluated through simulation analysis and comparison [73]. In order to adapt to dynamically changing user behaviors and demands, Gou and Wu (2022) considered users' historical behaviors and current transmission requirements. Then, they introduced a dynamic personalized data transmission model based on recurrent neural networks and attention mechanisms. Results indicated that this model outperformed other methods in terms of performance [74]. Thouti et al. (2022) suggested a framework based on the Convolutional Neural Network (CNN) to detect faults inside IoT devices in order to correctly discriminate between legal and illegitimate IoT devices. They also created a system capable of classifying and identifying IoT devices, in which CNNs demonstrated good classification and identification accuracy [75]. In conclusion, growing evidence suggests that merging neural networks and IoT can produce better results. In this work, IoT technology is also used to support the proposed ANN-CA model. IoT provides abundant data support and accurate input for the model, thus helping to better simulate the land use change. Real-time data collected by IoT sensors can help monitor land use and verify the accuracy of the model. For example, in the simulation of land use change, real-time monitoring of land use through IoT sensors can provide more accurate input data for ANN-CA model. In this way, ANN-CA model will be more accurate and reliable in simulating land use change. Meanwhile, IoT technology can also be used to verify the accuracy of ANN-CA model, and evaluate the simulation effect of the model by comparing it with real-time monitoring land use data.

The results show that the simulation accuracy of ANN-CA model in simulating land use change in 2020 is over 80 %, and the highest is 89.69 %. This result provides strong data support for urban planning and land management policies in County M area. Based on these high-precision land use prediction results, policy makers can formulate more scientific and reasonable land use strategies, optimize the allocation of land resources and promote the sustainable development of cities. Compared with the research conducted by Saputra and Lee in North Sumatra, Indonesia, this work has improved the simulation accuracy of the model, which may be due to the introduction of IoT technology for data collection and real-time monitoring. The application of IoT technology provides more real-time and accurate input data for the model, thus improving the accuracy of simulation. In addition, compared with the study of e Silva et al. in the semi-arid river basin in northeast Brazil, this work pays more attention to ecological protection and land use restrictions when considering the driving factors affecting land use change, which is reflected in the protection of ecological red line and basic farmland. Although this work has achieved good results in simulation accuracy, it also has some limitations. For example, this work mainly focuses on County M area, and the applicability of the model may need further verification in other areas or different types of urbanization. Future research can expand the scope of case studies and verify the applicability and effectiveness of the model in different regions.

In summary, the combination of neural network and IoT technology has broad application prospects in the field of land planning. IoT technology provides abundant data support and more accurate input for neural network model, thus improving the accuracy and reliability of land planning and land use change simulation. In a word, the technologies and models used in this work can help urban planning and land use to be more intelligent and sustainable, thus helping to realize the United Nations SDG 11 (sustainable cities and communities) and SDG 15 (protection of terrestrial ecosystems).

## 5 Conclusion

### 5.1 Research contribution

The research results and conclusions of this work mainly focus on the following aspects.

1. Land use prediction results: (1) By 2030, the construction land area of County M will continue to increase, while the agricultural land and woodland areas will continue to decrease, and the water area will remain basically stable. (2) From 2020 to 2030, the decrease of agricultural land and the increase of construction land decreased compared with 2010–2020, and the expansion rate of construction land slowed down slightly.
2. Suitability evaluation of urban development:
  (1) Through the evaluation of urban development suitability of County M, it is found that the suitability presents significant spatial differences within the county. The central area is more suitable for development, while the surrounding areas are relatively less suitable. (2) This suitability assessment provides decision support for urban planning and helps to guide the rational distribution of land resources and the orderly development of urban space.
3. Model accuracy verification:
  (1) With the reasonable setting and processing of model parameters and data, the simulation accuracy of ANN-CA model in simulating land use change in 2020 is over 80 %, and the highest is 89.69 %. (2) The analysis of Kappa coefficient shows that all land types and comprehensive kappa coefficient are greater than 0.75, which shows that the model has high accuracy and good effect in simulating land use change.
4. Research contributions: (1) This work constructs a comprehensive evaluation system of urban development suitability, which considers many factors and provides scientific basis and strategic guidance for future sustainable urban development. (2) By integrating technologies such as IoT, AI and cellular automata, the work provides an innovative and intelligent method to cope with the complexity and challenges brought by urbanization.

To sum up, this work not only provides quantitative prediction and evaluation results for land use planning and urban development of County M, but also provides strong technical support and practical significance for achieving the goal of sustainable urban development through high-accuracy model verification. The research results emphasize the importance of applying advanced technology in urban planning and development, and provide valuable experience and reference for future urban planning and development practice.

The novelty of this work lies in the comprehensive application of IoT technology and ANN-CA model to analyze and evaluate the suitability of land spatial planning and urban development. Firstly, the work uses the data and intelligent support provided by the IoT technology to optimize the decision-making of land spatial planning, involving resource utilization, environmental management and urban development. Secondly, through the ANN-CA model and the concept of “double evaluation”, the index system of urban development suitability evaluation is established, including permanent basic farmland, ecological red line and current construction land. The model shows high accuracy and reliability in simulating the land use evolution process, and provides an accurate prediction for the land use situation in M County in 2020. Finally, based on the established suitability evaluation index system, the suitability of urban development in M county is evaluated, which reveals the spatial differences of urban development within the county, provides valuable guidance for decision makers and researchers, and promotes orderly urban development and sustainable prosperity. On the whole, this work provides innovative methods and feasible guidance for the decision-making of land spatial planning and urban development by combining the IoT technology and ANN-CA model, which has certain theoretical and practical significance.

### 5.2 Future works and research limitations

There are still certain limitations. First, this work solely focuses on County M as the study subject, which may restrict its generalizability and applicability. Future research could encompass a broader range of case studies to validate the reliability and suitability of the model. Furthermore, while this work provides a comprehensive framework for appropriateness assessment, the practical practicality and operability of its application must be examined. Following study might look into how to effectively transform research findings into practical planning decisions, as well as how to continuously optimize and refine the assessment system through real-world application.

## Data availability statement

Data will be made available on request.

## CRediT authorship contribution statement

**Zhaoliang Nie:** Writing – original draft, Visualization, Validation, Software, Formal analysis, Data curation, Conceptualization.

## Declaration of competing interest

The authors declare that they have no known competing financial interests or personal relationships that could have appeared to influence the work reported in this paper.
